# A Forward Identification Method for High-Temperature Stress–Strain Curves of 7075 Aluminum Alloy Sheet Considering the Necking Stage

**DOI:** 10.3390/ma15207093

**Published:** 2022-10-12

**Authors:** Dan Yao, Yongchuan Duan, Yingping Guan, Shilong Pu

**Affiliations:** 1 Key Laboratory of Advanced Forging & Stamping Technology and Science, Yanshan University, Ministry of Education of China, Qinhuangdao 066004, China; 2 School of Mechanical Engineering, Yanshan University, Qinhuangdao 066004, China

**Keywords:** 7075 aluminum alloy, diffuse necking, forward identification method, viscoplastic constitutive model

## Abstract

The necking phenomenon of metal sheet under high temperatures is serious and continues over a longer duration. It is difficult to describe the high-temperature mechanical properties of materials only on the basis of hardening behavior before necking. To obtain the high-temperature stress–strain curve considering diffuse necking stage, a forward identification method based on strain measurement is proposed in this study. Here, the strain field of the minimum cross-section in the necking region of the specimen is obtained using a DIC (digital image correlation) measurement technique, and the average axial true stress–strain curve is calculated. Then, the average axial true stress–strain curve is modified using the modified Bridgeman formula. Taking 7075 aluminum alloy as an example, the high temperature equivalent stress–strain curve considering the diffuse necking stage is obtained. Compared with the traditional method, the maximum effective strain range is expanded from 0.05 to 0.8 due to the consideration of the necking stage. The obtained curve is characterized by a coupled viscoplastic–damage constitutive model and embedded in ABAQUS through the user subroutine VUMAT to simulate the hot tensile process. The relative error of force–displacement between the simulation and the experiment was 2.4%, validating the ability of the presented method. This study provides theoretical guidance and a scientific basis for the application and forming control of hot stamping processes.

## 1. Introduction

Due to improved ductility at high temperatures, hot forming processes of metal materials have been widely employed to manufacture components with complex shapes [1,2,3]. The use of forming processes using finite element analysis (FEA) can reduce the time and cost required for fabrication and improve product quality. To obtain reliable analysis results, accurate and reliable mechanical performance is essential. Compared with the mechanical behavior of cold-formed metals, the deformation of metal sheets such as aluminum alloy is greater during hot stamping. Hence, it is necessary to determine the flow curve in a large strain range.

Uniaxial tensile testing is an important mechanical experiment with simple operation and wide application. It is the main experimental method for determining the stress–strain relationship of materials [4]. The standard tensile test can only be used to obtain the accurate true stress–strain curve for the maximum uniform elongation. With the further stretching of the specimen, necking occurs, and the flow curve of this stage is difficult to determine, especially in the case of materials like 7075 aluminum alloy. The research of Lu et al. [5] and Wang et al. [6] indicated that 7075 aluminum alloy exhibits strong softening behavior under high-temperature conditions, and necking occurs early and continues for a longer period. The flow curve before necking is not sufficient to describe the high-temperature mechanical properties. For such materials, obtaining a complete flow curve is even more important.

To date, scholars have proposed some unconventional methods for obtaining the flow curve after necking [7,8,9]. One is the reverse identification method based on the finite element method. This method continuously corrects the stress–strain curve by means of the finite element model, so that the load–displacement curves of the simulation and the experiment gradually approach one another [10,11,12]. Pham et al. [13] proposed a hybrid method combining curve fitting and finite element inversion to identify the parameters of the hardening model over a large strain range. The curve fitting method maintains the accuracy of the hardening model before necking, and the finite element inverse method maintains the accuracy of the predicted curve after necking. Zhong et al. [14] proposed a method combining finite element analysis and hybrid particle swarm optimization to extract the yield curve of round rod after necking. The results indicated that the curve obtained using this method was reliable. Typically, the flow behavior of thermoformed materials is relatively complex, showing the coupled effects of work hardening, recovery, recrystallization, grain evolution, microstructure evolution, and damage [15,16,17]. Hence, the finite element inverse analysis method is difficult to perform. Another method is to obtain the local geometric contour parameters or local strain information of the necking region during stretching using image dynamic measurement technology, and directly calculate the stress and strain [18,19,20]. Paul et al. [21] carried out a finite element analysis of cylindrical specimen, calculated the equivalent strain and axial true stress according to the local axial strain in the necking region, and converted the axial true stress into equivalent stress by introducing an appropriate correction coefficient. Li et al. [22] proposed a method for obtaining the true stress–strain curve over a large strain range: DIC (Digital Image Correlation) was used to measure the cross-section area and strain field of the tensile specimen, and the true stress was calculated on the basis of the measured cross-section area and the load. However, due to the interference of the heating environment, dynamic images are difficult to obtain. Therefore, these two methods are usually applied in order to determine mechanical properties at room temperature, and are rarely applied under high-temperature conditions.

In this study, a forward identification method for obtaining the stress–strain curve considering the necking stage is proposed. With the assistance of strain measurement technology, the strain field distribution in the necking region of the specimen when subjected to hot tension can be obtained, and the average axial true stress–strain curve can be measured. Considering the change in stress state after necking, the average axial true stress–strain curve is modified using the modified Bridgeman formula, and the high-temperature equivalent stress–strain curve including the necking stage is obtained. Based on this method, the high-temperature equivalent stress–strain curve of 7075 aluminum alloy considering the necking stage is obtained. The obtained curves are characterized by a coupled viscoplastic–damage constitutive model and embedded in ABAQUS through a user subroutine VUMAT to simulate the hot tensile process. The good agreement between the numerical predictions and experimental measurements of force–displacement and geometrical size validate the effectiveness of the proposed method.

## 2. Materials and Methods

**Figure 1:**
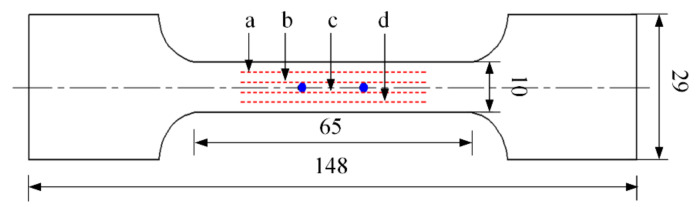
Hot tensile specimen of AA7075 alloy (mm).

7075 aluminum alloy exhibits strong softening behavior at high temperature, and the competition between work hardening and dynamic recovery is balanced at low strain. Meanwhile, the precipitation of the second-phase particles results in the early occurrence of damage, and damage continues for a longer period. Compared with other materials, the necking of 7075 aluminum alloy occurs earlier under high temperatures, and undergoes a long necking stage before fracture. In this context, 7075-T6 aluminum alloy with a thickness of 2 mm was selected as the research object in this study. The chemical composition is shown in Table 1. The original sheet was machined along the rolling direction via wire electrical discharge machining, and the dimensions of the hot tensile test specimens were adopted with reference to the international standard ISO 6892-2:2011, as shown in Figure 1.

**Figure 2:**
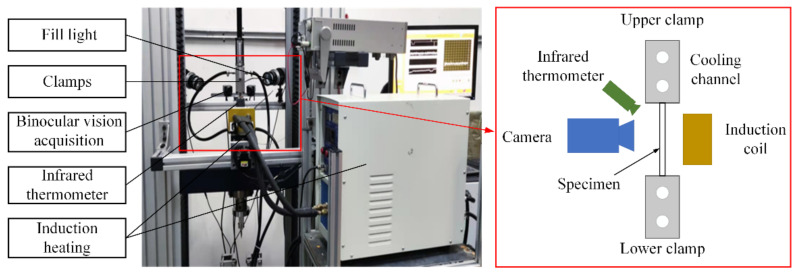
Experimental platform of the hot tensile tests.

To measure the high-temperature mechanical properties of 7075 aluminum alloy, hot tensile tests were carried out at different temperatures (350 °C, 400 °C, 450 °C) and strain rates (0.001 s^−1^, 0.01 s^−1^, 0.1 s^−1^). All tests were conducted on a 10 kN hydraulic universal testing machine equipped with a customized induction heating system, an infrared temperature measurement system, and a DIC system, as shown in Figure 2. The induction heating system was used for heating, and the infrared thermometer was used for temperature control. The emissivity of the coating for different temperatures was measured using a K-type thermocouple attached to the center of the specimen. The DIC system was used to perform dynamic image acquisition. Before the experiment, uniform black and white speckles were sprayed onto the surface of the specimen in advance. Two cameras (MER-030-120-GM/C-P) were used to record images, and the images were analyzed using VIC-3D software to determine the displacement field and strain field.

**Figure 3:**
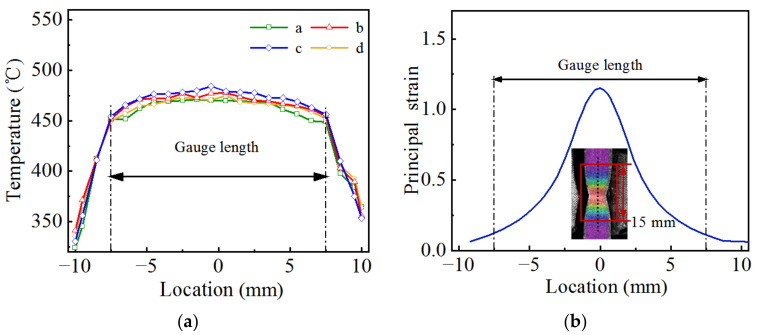
Temperature field and strain field of the specimen s urface at the moment of fracture: (**a**) temperature field; (**b**) strain field.

To determine the range of the uniform temperature section of the specimen during the hot tensile test, an infrared camera (American FLIR professional infrared thermal imager T460) was used to obtain the temperature field on the specimen surface at the moment of fracture, and the longitudinal temperature field at the −3, −1, 1 and 3 mm positions in the width direction (a, b, c and d in Figure 1) were extracted, as shown in Figure 3a. The difference in temperature of the original gauge length (15 mm) was controlled to 30 ℃ at the end of stretching. Figure 3b shows the strain field on the specimen surface after the tensile test, at which point the deformation was mainly concentrated in the range of 15 mm at the center of the specimen. The strain and temperature fields indicate that it is reasonable to set the gauge length to 15 mm.

**Figure 4:**
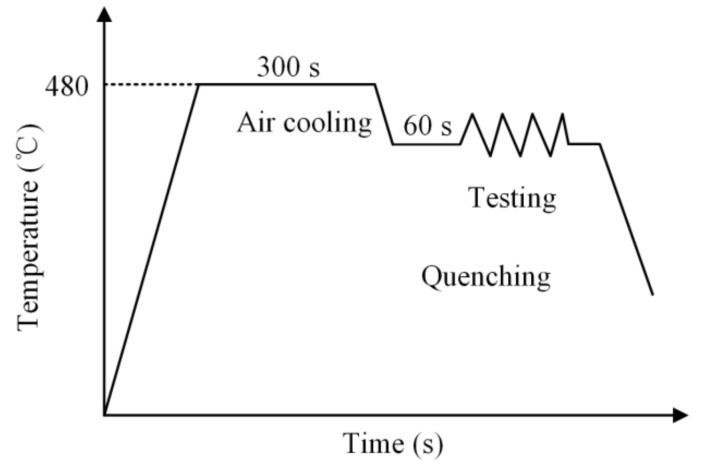
Schematic diagram of the hot tensile test.

Figure 4 shows the schematic diagram of the hot tensile test. Initially, the specimens were heated to 480 °C and kept at this temperature for 300 s to ensure a fully solid solution. Subsequently, the specimens were cooled to the target tensile temperature by means of air cooling, and held for 60 s to achieve a uniform temperature. Finally, the specimens were stretched to fracture at the target strain rates (0.001 s^−1^, 0.01 s^−1^, and 0.1 s^−1^).

## 3. Forward Identification Method for High Temperature Stress–Strain Curves Considering the Necking Stage

**Figure 5:**
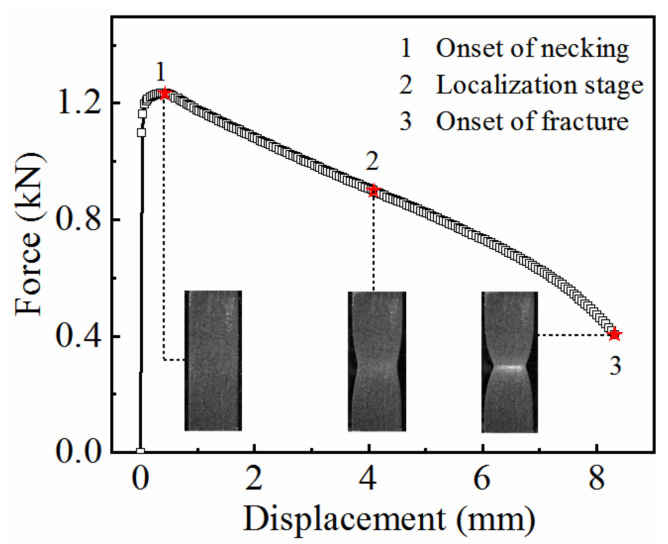
Force–displacement curve of 7075 aluminum alloy at a temperature of 350 °C and a strain rate of 0.01 s^−1^.

Figure 5 shows the force–displacement curve at a temperature of 350 ℃ and a strain rate of 0.01 s^−1^. The necking phenomenon of 7075 aluminum alloy occurred during the initial stage of deformation, and began almost as soon as it has entered the plastic stage, see label “1” in Figure 5. In the stretching process from label “1” to label “3”, the material experienced a long diffuse necking stage. Based on the uniform deformation theory, the force–displacement curve was transformed into the true stress–strain curve, as follows:

(1) $$\varepsilon =\ln\left(1+\frac{\Delta l}{l_{0}}\right)$$

(2) $$\sigma =\frac{F}{A_{0}}\left(1+\frac{\Delta l}{l_{0}}\right)$$

where ε and σ are true stress and true strain, respectively, *l*_0_ is the initial gauge length, Δl represents the instantaneous elongation of gauge length, *A*_0_ is the initial cross-section area of gauge length, and *F* is the load.

**Figure 6:**
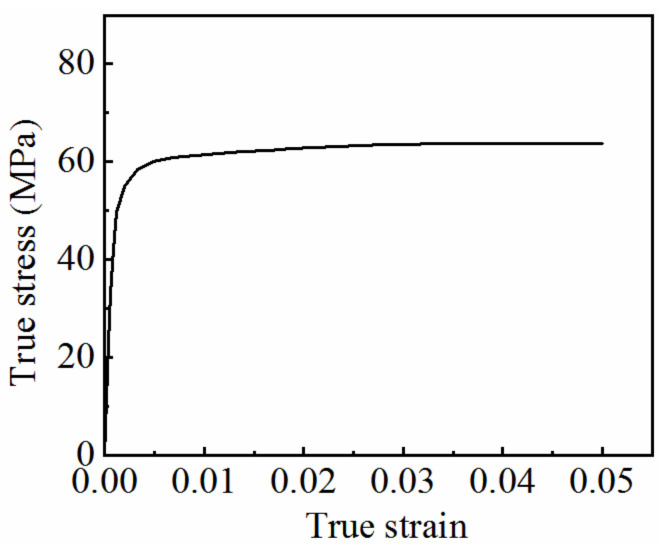
True stress–strain curve of 7075 aluminum alloy at 350 °C and strain rate of 0.01 s^−1^ obtained by uniform deformation calculation method.

Because the deformation of the material in the necking stage is non-uniform, Equations (1) and (2) are only applicable for calculating the true stress–strain curve before necking. As shown in Figure 6, the maximum strain that can be calculated is only 0.05. The curve is not able to fully reflect the mechanical properties of the material under high-temperature conditions. Therefore, the calculation range of the true stress–strain curve must be extended.

**Figure 7:**
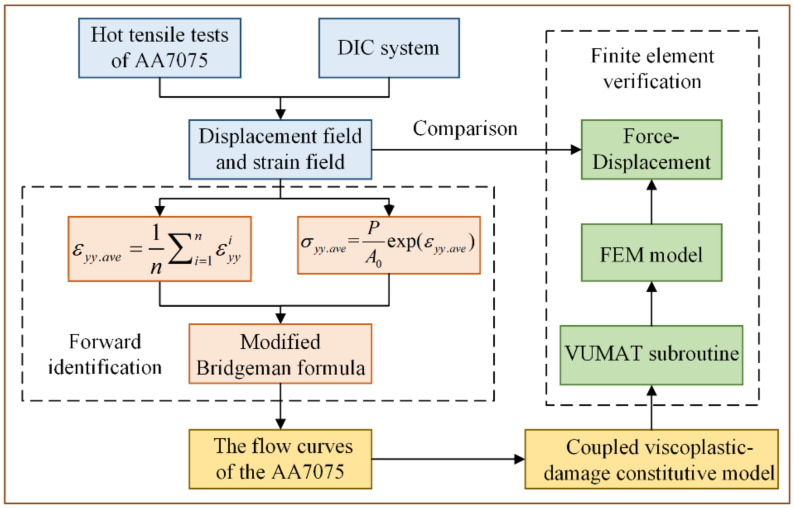
Flow chart of the forward identification method.

To obtain the high-temperature stress–strain curve including the diffuse necking stage, a forward identification method is established in this study by combining strain measurement technology with the modified Bridgeman formula. The specific process is shown in Figure 7. Taking the hot tensile test at 350 °C and a strain rate of 0.01 s^−1^ as an example, the calculation process is described in detail.

### 3.1. Calculation Method of Average True Stress–Strain Curve Based on Strain Measurement Technology

**Figure 8:**
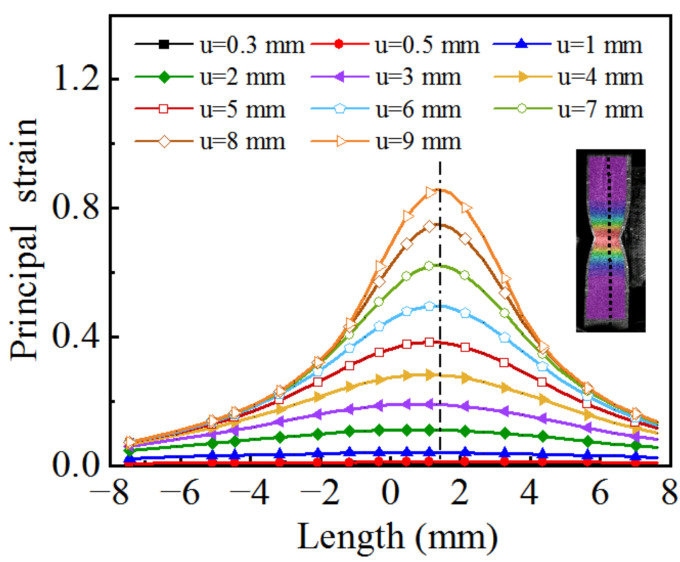
Distribution of principal strain in the longitudinal direction.

Based on the strain field on the specimen surface obtained by DIC, a straight line is drawn along the length direction of the specimen, and the local principal strain of each point on the line at different tensile strokes is extracted, as shown in Figure 8, where u represents the tensile stroke of the testing machine. The results indicate that when the tensile stroke is 2 mm, the distribution of the principal strain along the length direction is localized, and the necking phenomenon becomes more obvious with increasing deformation.

**Figure 9:**
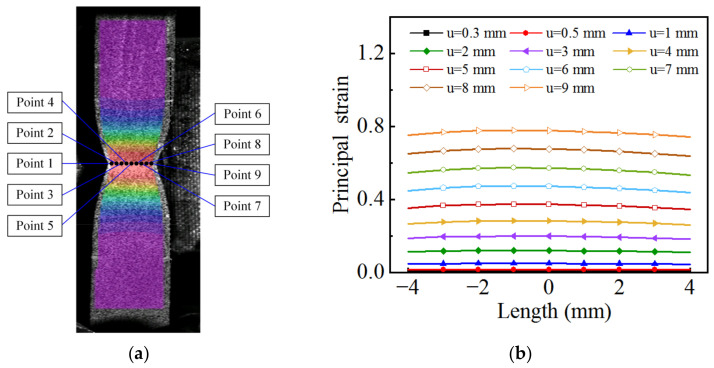
Principal strain distribution on the transverse line of the minimum cross-section: (**a**) location of measuring point; (**b**) principal strain distribution.

A transverse line is drawn at the minimum cross-section of the necking region, and the local principal strain of each point on the line at different tensile strokes is extracted, as shown in Figure 9, where u represents the tensile stroke of the testing machine. The results show that although the fluctuation of the principal strain in the transverse direction is obviously smaller than that in the longitudinal direction, the fluctuation still exists. To reduce the influence of non-uniform strain distribution, the average value of local principal strain at each point is taken as the average axial true strain, which can be expressed as:

(3) $$\varepsilon_{yy.ave}=\frac{1}{n}\sum_{i=1}^{n}\varepsilon_{yy}^{i}$$

where $\varepsilon_{yy}$ is the local principal strain of each point, and *n* is the number of points.

The relationship between the average axial true strain of the minimum cross-section and its area can be calculated as follows [8]:

(4) $$\varepsilon_{yy.ave}=\ln(\frac{A_{0}}{A})$$

where *A* is the real-time area of the minimum cross-section.

Therefore, the real-time area of the minimum cross-section can be expressed as follows:

(5) $$A=\frac{A_{0}}{\exp(\varepsilon_{yy.ave})}$$

The average axial true stress of the minimum cross-section can be expressed as follows:

(6) $$\sigma_{yy.ave}=\frac{F}{A}=\frac{F}{A_{0}}\exp(\varepsilon_{yy.ave})$$

**Figure 10:**
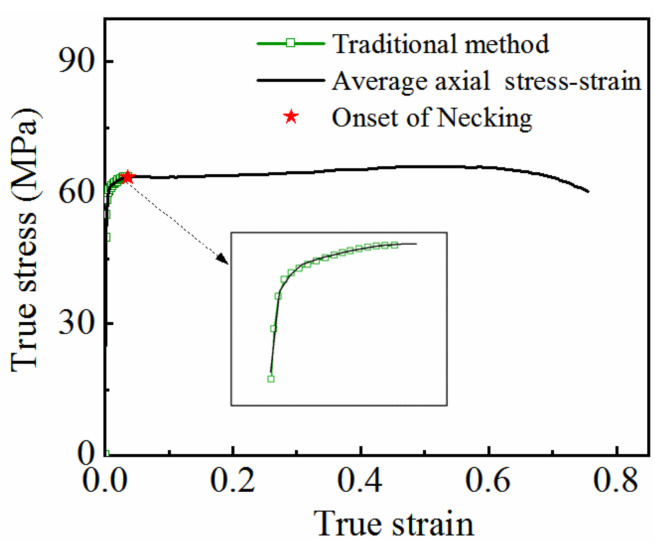
Axial average true stress–strain curve based on strain measurement technology.

The average axial true stress–strain curve obtained using the strain measurement technology is shown in Figure 10. It can be seen from the figure that the average axial true stress–strain curve before necking coincides with the true stress–strain curve obtained by the uniform deformation calculation method, indicating the reliability of strain measurement technology. 

### 3.2. Modification of Equivalent Stress–Strain Curves

For the uniaxial tensile specimen of the sheet, the local deformation region of the specimen after necking is no longer in the uniaxial stress state, but in the two-dimensional plane stress state. Therefore, the average axial true stress–strain obtained using DIC measurement technology is not equivalent to the equivalent stress–strain, and further modification is needed. In this study, the modified Bridgeman formula [8] is adopted to perform this modification:

(7) $$\sigma_{eq}=\frac{\sigma_{yy.ave}}{\left(1+\frac{2R}{a})\ln(1+\frac{a}{2R}\right)}$$

(8) $$\frac{a}{R}=1.1(\varepsilon_{eq}-\varepsilon_{p\max})$$

where $\varepsilon_{p\max}$ is the strain at the onset of necking, $\varepsilon_{eq}$ is the equivalent strain, which can be approximately replaced by the average axial true strain $\varepsilon_{yy.ave}$, a is the radius of minimum cross-section, and *R* is the curvature radius of outer contour.

**Figure 11:**
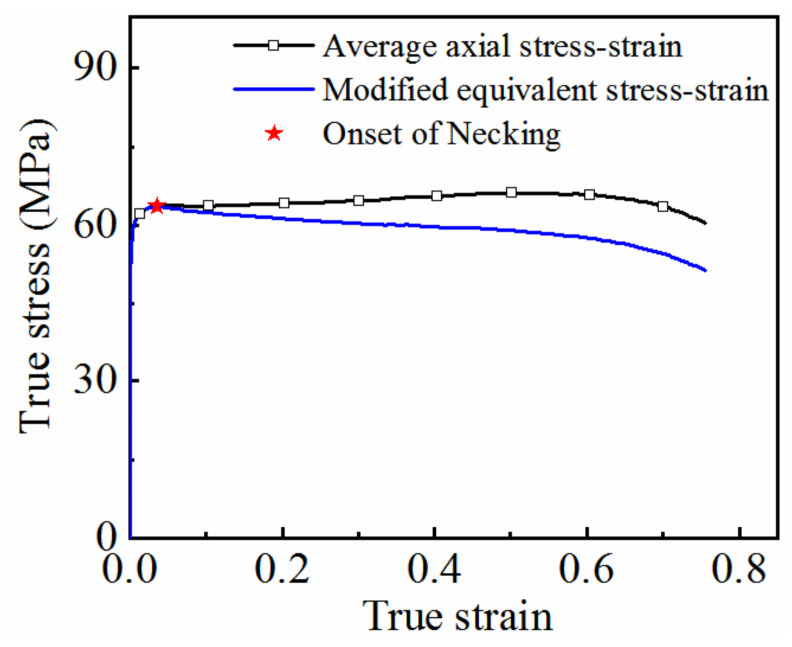
Modified equivalent stress–strain curve.

The modified equivalent stress–strain curve is shown in Figure 11, indicating that the modified equivalent stress is lower than the average axial true stress.

### 3.3. High-Temperature Flow Curve of 7075 Aluminum Alloy

**Figure 12:**
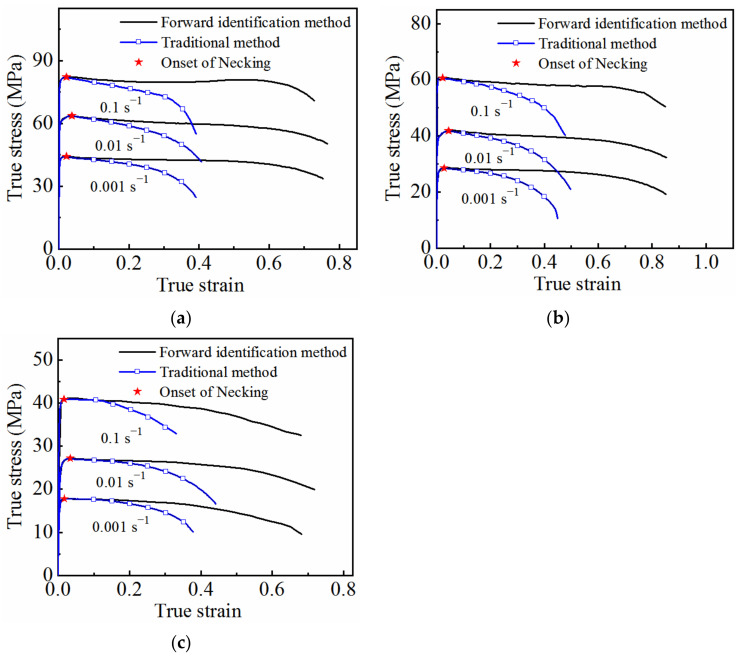
Modified equivalent stress–strain curve of 7075 aluminum alloy under different deformation conditions: (**a**) 350 °C; (**b**) 400 °C; (**c**) 450 °C.

Based on the above method, the flow curves of 7075 aluminum alloy under different deformation conditions are identified, as shown in Figure 12. During the initial stage of thermal deformation, the stress increases rapidly with increasing strain. After the material yields, the softening effect caused by dynamic recovery is generated, which offsets part of the work hardening. Owing to the strong dynamic softening effect of the aluminum alloy at high temperatures, the effects of work hardening and softening are essentially balanced at lower strain values (i.e., less than 0.05), and the stress reaches its peak. Meanwhile, the cavities caused by the second phase particles grow with increasing deformation during the plastic deformation process, resulting in a decrease in the carrying capacity of the material, and diffuse necking occurs.

The curve determined using the forward identification method was compared with that obtained using the uniform deformation calculation method, as shown in Figure 12. The two curves coincide before necking. The curve obtained using the uniform deformation calculation method is only valid before necking, and the effective strain range is about 0–0.05 (see Figure 12, star). When considering necking, the effective strain range obtained using the forward identification method proposed is about 0–0.8, which is significantly higher than the strain range before necking.

## 4. Result and Discussion 

Based on the modified high-temperature equivalent stress–strain, a hot tensile finite element model under different deformation conditions is established. By comparing the force–displacement curves obtained from the simulation with those obtained from the experiment, as well as the geometrical size of the fractured cross-sections, it was possible to verify the accuracy of the forward identification method.

### 4.1. Establishment of Coupled Viscoplastic–Damage Constitutive Model

The coupled viscoplastic–damage constitutive model describes the evolution of internal structure and inelastic strain through a set of interrelated evolution equations of internal variables, and is widely used to describe the high-temperature deformation behavior of metal materials:

(9) $$\left\{\begin{array}{l} {\dot{\varepsilon }}_{p}={\left(\frac{\sigma /(1-D)-H-k}{K}\right)}^{n_{1}}\\ H=B{\bar{\rho }}^{1/2}\\ \dot{\bar{\rho }}=A(1-\bar{\rho })\left|{\dot{\varepsilon }}_{p}\right|-C{\bar{\rho }}^{n_{2}}\\ \dot{D}=d_{1}\left(1-D\right){\dot{\varepsilon }}_{p}^{d_{2}}+\frac{D_{1}{\dot{\varepsilon }}_{p}^{D_{2}}\cosh(d_{3}\varepsilon_{p})}{{\left(1-D\right)}^{D_{3}}}\\ \sigma =E(1-D)(\varepsilon_{T}-\varepsilon_{p}) \end{array}\right.$$

where ${\dot{\varepsilon }}_{p}$ is the plastic strain rate, *n*_1_ is the viscosity exponent, *H* is the hardening constant, *K* and *k* are the resistance coefficient and initial yield stress, respectively, $\bar{\rho }$ is the normalized dislocation density, *D* is the damage variable, *E* is the elasticity modulus, $\varepsilon_{T}$ and $\varepsilon_{p}$ are the total strain and equivalent plastic strain, respectively, and *A*, *B*, *C*, *D*_1_, *D*_2_, *D*_3_, *d*_1_, *d*_2_, *d*_3_ and *n*_2_ are material constants.

To improve the ability of the model to describe the mechanical properties of materials at different temperatures, the material parameters *A*, *B*, *C*, *D*_1_, *D*_2_, *D*_3_, *K*, *k* and *E* were set as temperature-related parameters, as follows:

(10) $$\left\{\begin{array}{l} A=A_{0}\exp(\frac{Q_{A}}{R_{g}T})\\ C=C_{0}\exp(-\frac{Q_{C}}{R_{g}T})\\ B=B_{0}\exp(\frac{Q_{B}}{R_{g}T})\\ K=K_{0}\exp(\frac{Q_{K}}{R_{g}T})\\ k=k_{0}\exp(\frac{Q_{k}}{R_{g}T})\\ D_{1}=D_{10}\exp(\frac{-Q_{D_{1}}}{R_{g}T})\\ D_{2}=D_{20}\exp(\frac{Q_{D_{2}}}{R_{g}T})\\ D_{3}=D_{30}\exp(\frac{Q_{D_{3}}}{R_{g}T}) \end{array}\right.$$

where *A*_0_, *C*_0_, *B*_0_, *D*_10_, *D*_20_, *D*_30_, *K*_0_ and *k*_0_ are material constants, *Q_A_*, *Q_B_*, *Q_C_*, *Q_D_*_1_, *Q_D_*_2_, *Q_D_*_3_, *Q_K_* and *Q_k_* are the activation energy of the corresponding material constants, *R*_g_ is the gas constant, and *T* is the temperature (Kelvin).

Owing to several material parameters of the model, and the high degree of coupling between the equations, solving it directly using analytical methods is difficult. Optimization algorithms are usually used to identify the optimal solution of these parameters. In this study, a genetic algorithm was adopted to determine the material parameters of the model using a combination of MATLAB and the optimization software I-SIGHT. The objective function can be expressed as follows:

(11) $$f(x)=\frac{1}{M}\sum_{j=1}^{M}\left\{\frac{1}{N_{j}}\sum_{i=1}^{N_{j}}{\left(\ln\frac{\sigma_{ij}^{c}}{\sigma_{ij}^{e}}\right)}^{2}\right\}$$

where *f*(*x*) represents the difference between the experimental stress and model stress, *x* is the material parameter in the constitutive model, *M* is the number of curves; *N* is the number of experimental points in each curve; $\sigma_{ij}^{e}$ and $\sigma_{ij}^{c}$ are the experimental and calculated stress of the data points, respectively.

Based on the established objective function, the parameters were optimized using the stress–strain data for 7075 aluminum alloy. The number of experimental curves was nine, and 20 data points were extracted at equal intervals from each curve. Population size, number of generations, crossover rate, and mutation probability rate were set to 200, 1000, 0.8 and 0.05, respectively. The optimization results are shown in Table 2.

**Figure 13:**
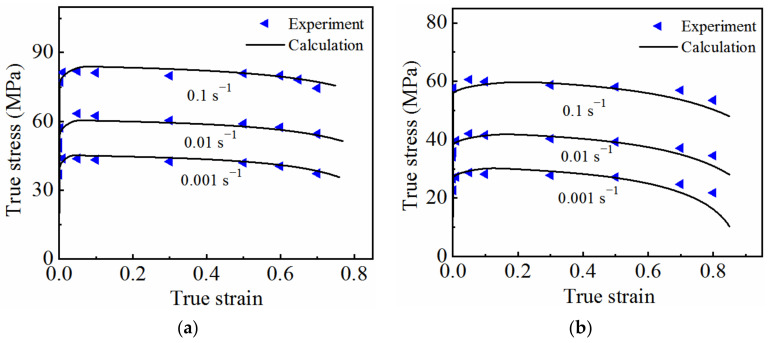

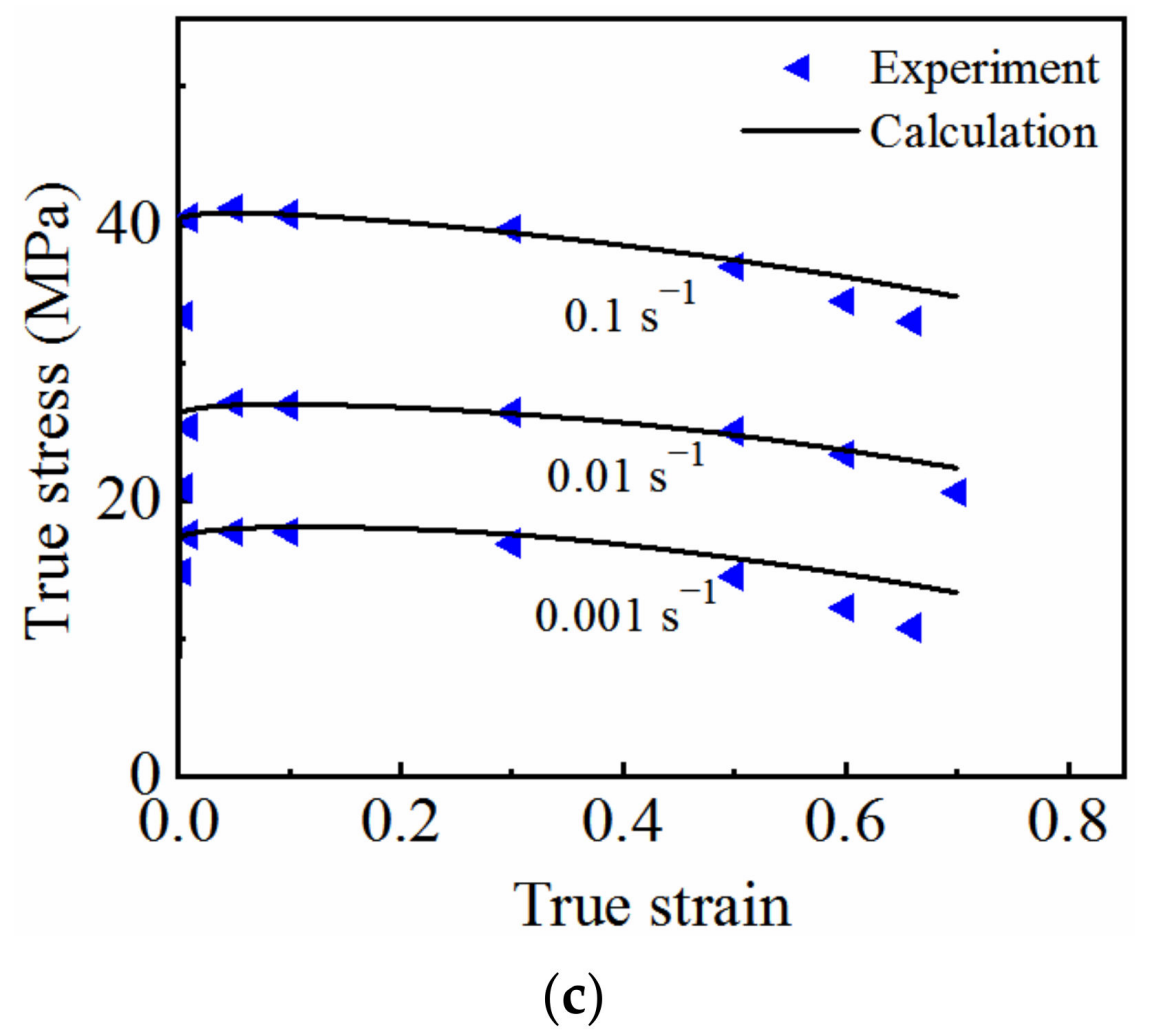
Comparison between the experimental results and the calculated results under different deformation conditions: (**a**) 350 °C; (**b**) 400 °C; (**c**) 450 °C.

The optimized parameter values were brought into the model to calculate the stress–strain curves under different deformation conditions, and the results were compared with the experimentally determined ones, as shown in Figure 13. The experimental results (symbols) were in good agreement with the calculated results (solid lines), indicating that the established coupled viscoplastic–damage constitutive model is able to correctly describe the high-temperature flow behavior of 7075 aluminum alloy.

### 4.2. Finite Element Simulation of Hot Tensile Testing

**Figure 14:**
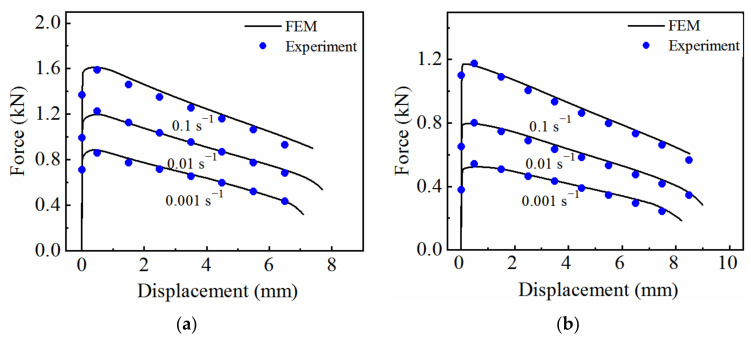

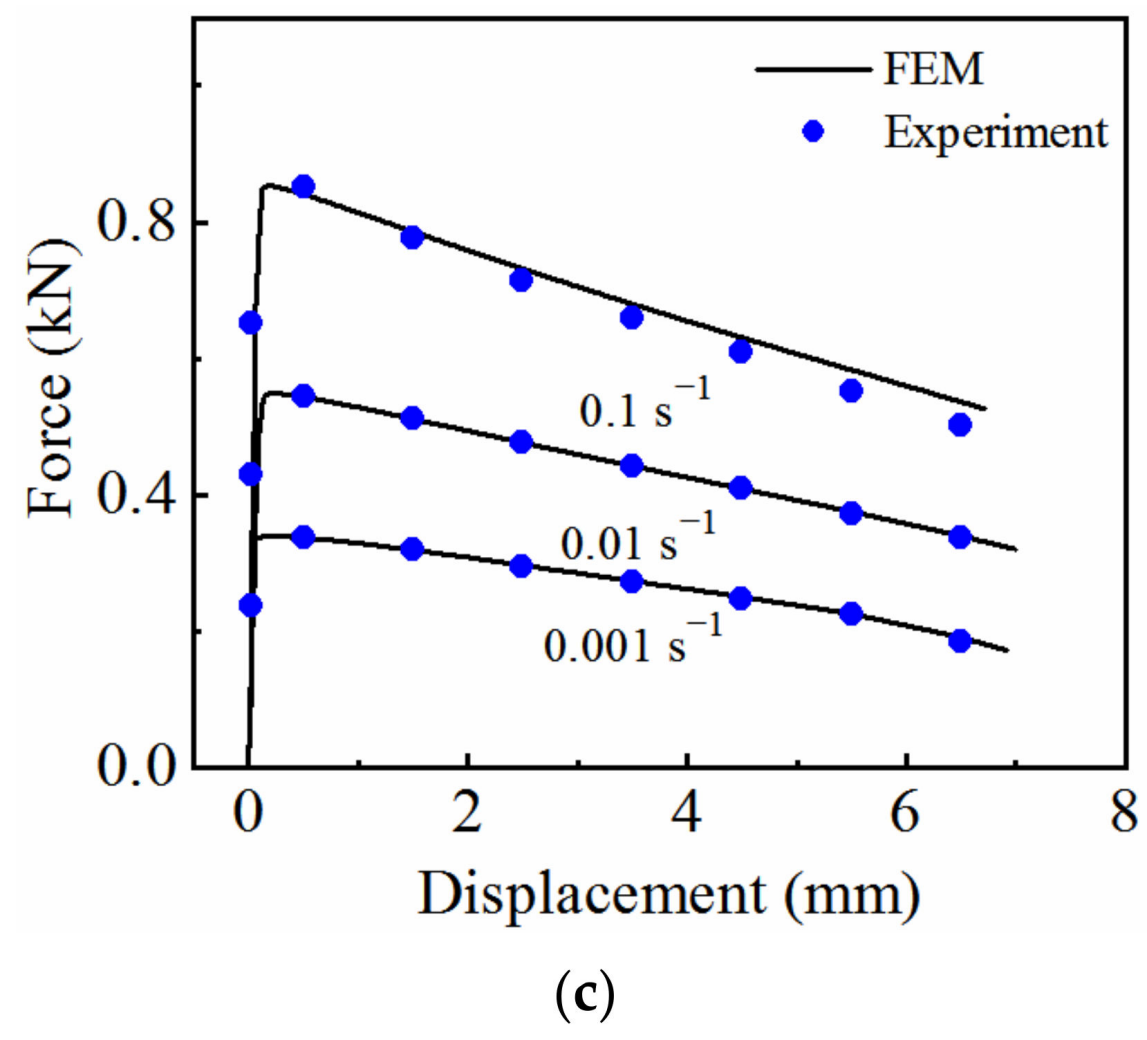
Comparison of force–displacement curves obtained from the simulation and the experiment under different deformation conditions: (**a**) 350 °C; (**b**) 400 °C; (**c**) 450 °C.

A user-defined material subroutine VUMAT of the coupled viscoplastic–damage constitutive model was developed, and a finite element model of the hot tensile process was established in ABAQUS. The temperature in the gauge length area was set as the deformation temperature, and the rest were set at room temperature. The mesh size (0.5 × 0.5 × 0.5) was refined in the gauge length region to improve the accuracy of the analysis, and the coarse mesh was refined in the remaining regions (1.5 × 1.5 × 0.5) to save computation time. To avoid the influence of mesh size, the same element type (C3D8T) and size were adopted for all models. The boundary conditions corresponding to the experiment were applied, i.e., one end was fixed, and the other end was subjected to displacement constraints. Figure 14 shows a comparison of the force–displacement curves obtained from the simulation (solid curves) with the experimentally determines ones (symbols). It can be seen that good agreement was achieved, with a relative error of 2.4%, indicating that the stress–strain curve determined using the forward identification method is able to accurately describe the hot tensile process of 7075 aluminum alloy.

**Figure 15:**
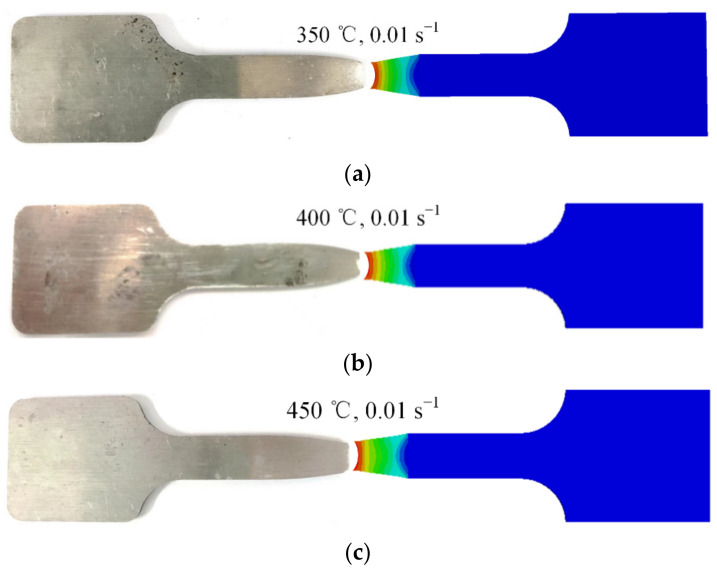
Comparison of specimen geometrical size obtained by simulation and experiment: (**a**) 350 °C, 0.01 s^−1^; (**b**) 400 °C, 0.01 s^−1^; (**c**) 450 °C, 0.01 s^−1^.

Figure 15 compares the simulated and experimental geometrical size of the specimens stretched to fracture. An obvious necking phenomenon can be observed in both the simulations and the experiments, and the geometrical size of the necking region obtained by simulation is consistent with that shown by the experiment. The results indicate that the finite element model is able to accurately describe the necking phenomenon of 7075 aluminum alloy during the process of hot tensile testing.

## 5. Conclusions

In this study, a forward identification method for obtaining equivalent stress–strain curves including diffuse necking stage is proposed. The method is applied to 7075 aluminum alloy, and the equivalent stress–strain curves under different deformation conditions are obtained. The following conclusions can be drawn:

(1) With the aid of DIC measurement technology, the strain field distribution on the specimen surface during hot tensile process is obtained. Based on the local strain field of the minimum cross-section of the necking region, the average axial true stress–strain curve is obtained.
(2) Considering the change in the stress state after necking, the average axial true stress–strain curve is modified using the modified Bridgeman formula, and the equivalent stress–strain curve including the diffuse necking stage is obtained. Compared with the uniform deformation calculation method, the range of the stress–strain curve is significantly increased, and the effective strain range is expanded from 0–0.05 to 0–0.8, indicating that this method can be used to effectively determine the stress–strain curve in a large strain range.
(3) Based on the high-temperature equivalent stress–strain curve obtained using the forward identification method, a coupled viscoplastic–damage constitutive model is established, and embedded into the finite element model for the purposes of simulation analysis. The relative error of simulated and experimental force–displacement curves is 2.4%, and the geometrical size of the necking region obtained by simulation is consistent with the experiment, indicating that the obtained curve is well able to describe the high-temperature flow behavior of 7075 aluminum alloy.

## Figures and Tables

**Table 1:** Chemical composition of AA7075 sheet (weight %).

Zn	Mg	Cu	Cr	Fe	Mn	Zr	Ti	Si	Al
5.48	2.3	1.52	0.21	0.16	0.03	0.01	0.03	0.11	>90.1

**Table 2:** Parameters in the constitutive model of 7075 aluminum alloy.

Parameter	Result	Parameter	Result	Parameter	Result
*K*_0_ (MPa)	3.2717	*D*_10_ (MPa)	2.462	*A*_0_ (MPa)	2.2171 × 10^−8^
*Q*_K_ (J·mol^−1^)	1.7592 × 10^4^	*Q*_D1_ (J·mol^−1^)	2.2121 × 10^4^	*Q*_A_ (J·mol^−1^)	1.0330 × 10^5^
*k*_0_ (MPa)	4.4603 × 10^−7^	*D*_20_ (MPa)	0.3090	*n*_2_	8.41
*Q*_k_ (J·mol^−1^)	8.9094 × 10^4^	*Q*_D2_ (J·mol^−1^)	0.5539 × 10^4^	*d*_1_	0.0831
*B*_0_ (MPa)	0.1528	*D*_30_ (MPa)	0.3211	*d*_2_	2.6079
*Q*_B_ (J·mol^−1^)	2.2869 × 10^4^	*Q*_D3_ (J·mol^−1^)	5.3223 × 10^3^	*d*_3_	3.3734
*C*_0_ (MPa)	3.1797 × 10^3^	*E*_0_ (MPa)	22.2232	*n*_1_	5.13
*Q*_C_ (J·mol^−1^)	1.2463 × 10^4^	*Q*_E_ (J·mol^−1^)	3.9548 × 10^4^		

## Data Availability

Not applicable.
